# Treatment effects of the Jasper Jumper and the Bionator associated with fixed appliances

**DOI:** 10.1186/s40510-014-0054-9

**Published:** 2014-09-02

**Authors:** Leniana Santos Neves, Guilherme Janson, Rodrigo Hermont Cançado, Karina Jerônimo Rodrigues Santiago de Lima, Thaís Maria Freire Fernandes, José Fernando Castanha Henriques

**Affiliations:** Department of Orthodontics, Bauru Dental School, University of São Paulo, Bauru, Brazil; Department of Orthodontics, Ingá Faculty, Rodovia PR 317, n° 6114, Maringá, PR 87035-510 Brazil

**Keywords:** Activator appliances, Cephalometry, Orthodontic appliance design, Treatment outcome

## Abstract

**Background:**

The aim of this study was to evaluate the effects of Class II malocclusion treatment with the Jasper Jumper and the Bionator, associated with fixed appliances.

**Methods:**

The sample comprised 77 young individuals divided into 3 groups: Group 1 consisted of 25 patients treated with the Jasper Jumper appliance associated with fixed appliances for a mean period of 2.15 years; group 2 had 30 patients, treated with the Bionator and fixed appliances, for a mean treatment time of 3.92 years; and the control group included 22 subjects followed for a mean period of 2.13 years. The initial and final lateral cephalograms of the patients were evaluated. Intergroup comparison at the initial stage and of the treatment changes were performed by analysis of variance.

**Results:**

Their effects consisted in a restrictive effect on the maxilla, a slight increase in anterior face height, retrusion and extrusion of the maxillary incisors, labial tipping and protrusion of the mandibular incisors in both groups and intrusion with the Jasper Jumper appliance, maxillary molar distalization with the Jasper Jumper, extrusion and mesialization of the mandibular molars, both appliances provided significant improvement of the maxillomandibular relationship, overjet, overbite and molar relationship.

**Conclusions:**

The effects of both appliances in class II malocclusion treatment are similar; however, treatment with the Jasper Jumper was shorter than with the Bionator.

## Background

Class II malocclusion is characterized by an incorrect relationship between the maxillary and mandibular arches because of skeletal problems, dental problems, or a combination of both [1]. This malocclusion has been extensively studied regarding skeletal and dental characteristics, timing and methods of treatment [2-4]. The main reason for the extensive research on this particular type of malocclusion is its high frequency in the population [5]. This malocclusion is reported to constitute 12% to 49% of all orthodontic disorders [6]. A successful treatment of this malocclusion requires that the skeletal and dental basis of the disorder be carefully investigated [7,8]. The method of treatment is usually performed using orthopedic and orthodontic appliances.

Functional appliances that are an important part of orthodontic treatment demonstrate significant diversity in design, which could easily affect their acceptance by the patients. Although the Bionator or the Twin block is more acceptable as compared with activators, patients do not easily adapt to these appliances because of their large size and unfixed position in the mouth. Patient adaptation may vary regarding different functional appliances [9]. An ideal functional appliance should be comfortable for the patient, allow jaw movements, leave room for the tongue, provide skeletal rather than dental effects, and should be comfortable to be used in subjects with nasal obstruction [5].

Even though many previous studies focused on the clinical outcome of the Jasper Jumper, its treatment effects followed by fixed comprehensive therapy must be clarified [5,10–12]. No previous clinical investigation has evaluated the overall effects of a more comfortable functional appliance and compared it with the Bionator followed by fixed appliances in the treatment of class II malocclusion. Therefore, the purpose of this clinical study was to investigate the dentoskeletal changes in two groups of patients with class II division 1 malocclusion treated without extractions, either with the Jasper Jumper appliance followed by fixed comprehensive treatment or with the Bionator associated with fixed appliances.

## Methods

This study was approved by the Ethics in Research Committee of the University of São Paulo, and all subjects signed an informed consent. The study sample comprised 77 subjects (55 treated, 22 untreated). Fifty five patients who had been part of two prospective clinical trials in different time periods and were consecutively treated in the Orthodontic Department at Bauru Dental School, University of São Paulo were retrospectively evaluated in this clinical study. Sample selection was based exclusively on the initial anteroposterior molar relationship, regardless of any other dentoalveolar or skeletal cephalometric characteristics. All patients met the following inclusion criteria: (1) class II division 1 malocclusion with bilateral class II molar relationship (with a minimum severity of one-half class II molar relationship), (2) no craniofacial syndromes or systemic diseases, (3) no tooth agenesis or missing permanent teeth, and (4) mandibular arch showing minimal or no crowding.

The Jasper Jumper group (group 1) included 25 subjects (13 males; 12 females) treated with fixed appliances and the force modules of the Jasper Jumper appliance. All patients were in the early permanent dentition with all permanent first molars, and first and second premolars erupted. Their initial mean age was 12.72 years (SD = 1.21, range 10.32 to 14.84 years), and their final mean age was 14.88 (SD = 1.20, range 12.74 to 16.90 years), treated for a mean period of 2.15 years (SD = 0.30). These subjects had an initial ANB angle of 5.38° (SD = 2.87°) and a mean overjet of 6.24 mm (SD = 2.21 mm). This group was collected and treated by one operator (L.S.N.).

The Bionator followed by fixed appliances group (group 2) consisted of 30 subjects (16 males; 14 females). All patients were in the early permanent dentition with all permanent first molars, and first and second premolars erupted. This sample had an initial mean age of 11.31 years (SD = 1.19; range 9.27 to 14.00 years) and a final mean age of 15.23 (SD = 1.17, range 12.99 to 17.60 years), and was treated for a mean period of 3.92 years (SD = 1.62). These subjects had an initial ANB angle of 6.04° (SD = 2.09°) and a mean overjet of 8.42 mm (SD = 2.93 mm).

The control group (group 3) was obtained from the Longitudinal Growth Study files at Bauru Dental School, University of São Paulo. This group comprised 22 subjects (12 boys, 10 girls) with angle class II division 1 malocclusion with no orthodontic treatment, at an initial mean age of 12.67 years (SD = 0.75; range 11.21 to 13.98 years) and a final mean age of 14.80 (SD = 1.71), who were longitudinally followed for a mean period of 2.13 years (SD = 1.64). These subjects had an initial ANB angle of 4.11° (SD = 1.83°) and a mean overjet of 4.70 mm (SD = 1.60 mm).

**Figure 1:**
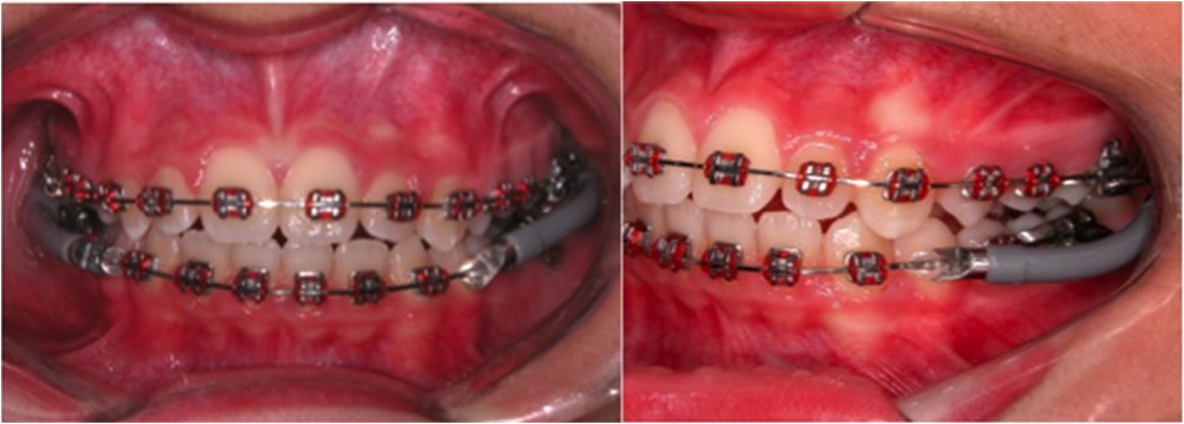
**Jasper Jumper appliance.**

**Figure 2:**
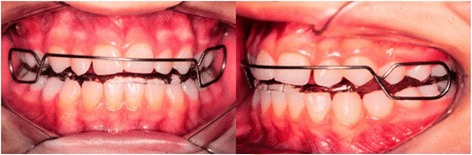
**Bionator appliance.**

Jasper Jumper (Figure 1) and Bionator (Figure 2) appliance designs were described in detail in previously published articles [13,14].

The mean treatment time with the Jasper Jumper was 7 months (range 3 to 12 months). After removal of the jumpers, the corrected anteroposterior relationship was retained with 5/16-inch class II elastics for a mean period of 4 months (range 1 to 8 months), with a daily recommended use of 14 h. After removal of the fixed orthodontic appliances, a Hawley plate was used in the maxillary arch and a canine to canine bonded retainer was used in the mandibular arch. A Bionator was also used at night for 12 months.

Group 2 patients underwent orthopedic treatment with the Bionator for a mean period of 1.27 years (SD = 0.53, range 0.38 to 2.31 years). After obtaining a normal anteroposterior relationship using this appliance, fixed standard edgewise appliances were installed. Leveling and alignment followed the usual sequence: 0.016 inch nickel-titanium, 0.018, 0.020, and 0.018 × 0.025-inch stainless steel archwires. Most patients (60%) used the headgear (KHG) for 14 h/day during fixed appliance treatment, as active retention. Others (40%) used class II elastics as active retention, for 14 h/day. These devices were used during 70% of the fixed appliance phase. After removal of the fixed orthodontic appliances, a Hawley plate was used in the maxillary arch and a canine-to-canine bonded retainer was used in the mandibular arch. All patients were treated non-extraction.

### Cephalometric analysis

**Figure 3:**
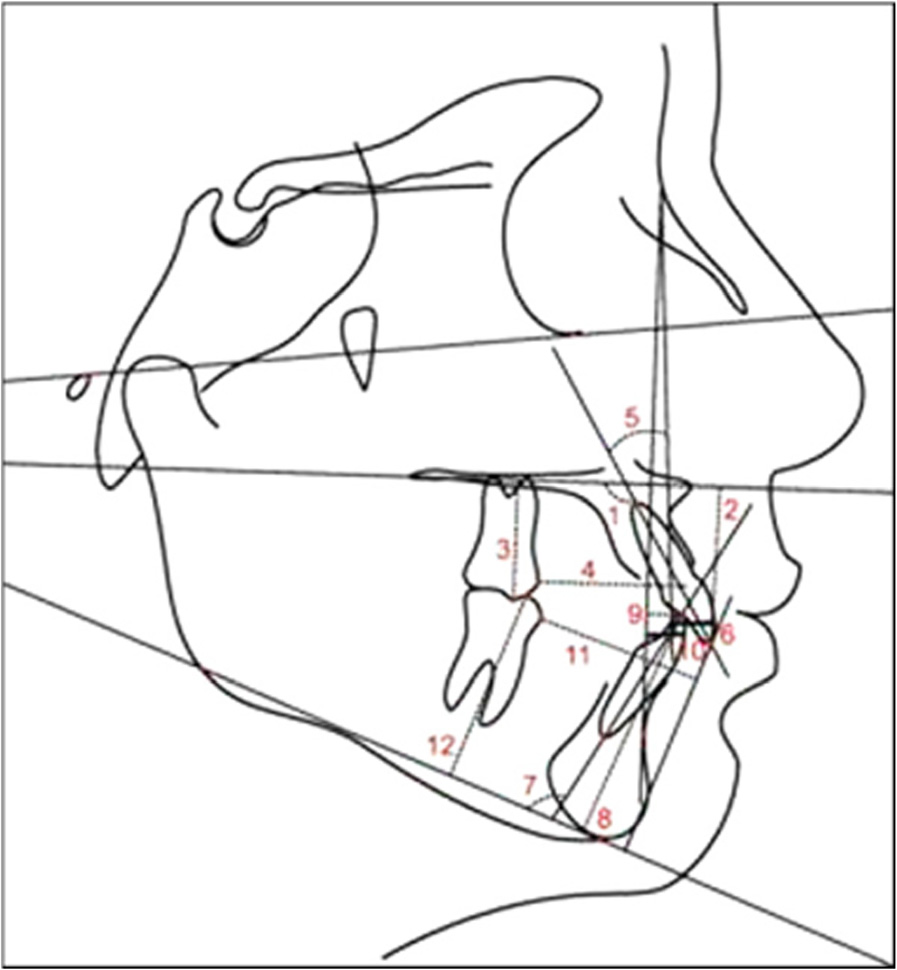
**Dentoalveolar cephalometric variables: 1, 1.PP; 2, 1-PP; 3, 6-PP; 4, 6-ANSperp; 5, 1.NA; 6, 1-NA; 7, IMPA; 8, 1-GoMe; 9, 1.NB; 10, 1-NB; 11, 6-Pogperp; 12, 6-GoMe.**

Lateral cephalograms of each patient were taken at the pre- and posttreatment stages (T1 and T2, respectively). Anatomic tracings of the lateral cephalograms and landmark locations were manually conducted and digitized (AccuGrid XNT, model A30TL.F, Numonics, Montgomeryville, PA, USA) by one investigator (L.S.N.). These data were then stored in a computer and analyzed with Dentofacial Planner software (version 7.02, Dentofacial Software, Toronto, ON, Canada). This software corrected the magnification factors (6%, 7.9%, and 9.8%) of the radiographic images. The less usual cephalometric variables are illustrated in Table 1 and Figure 3.

**Table 1:** **Definitions of abbreviations of the less usual cephalometric variables used**

	**Definition**
1.PP (°)	Angle formed by the maxillary incisor long axis and the palatal plane (PP)
1-PP (mm)	Linear distance from the maxillary central incisor edge projected perpendicularly to the PP
6-PP (mm)	Linear distance from mesiovestibular cusp of the maxillary first molar projected perpendicularly to the PP
6-ANSperp (mm)	Linear distance from the maxillary first molar mesial point to the ANSperp line (line perpendicular to palatal plane passing through anterior nasal spine)
1.NA (°)	Angle formed by the maxillary incisor long axis and the NA line
1-NA (mm)	Linear distance between the most anterior point of the maxillary central incisor and the NA line
IMPA (°)	Angle formed by the mandibular incisor long axis and the mandibular plane (GoMe)
1-GoMe (mm)	Linear distance between the mandibular incisor edge perpendicular to GoMe
A-Nperp (mm)	Linear distance from Point A to the Nperp line (line perpendicular to the Frankfort plane passing through point N)
Pog-Nperp (mm)	Linear distance from Pog to the Nperp line
1.NB (°)	Angle formed by the mandibular incisor long axis and the NB line
1-NB (mm)	Linear distance between the most anterior point of the mandibular central incisor and the NB line
6-Pogperp (mm)	Linear distance between the mandibular first molar mesial point to the Pog-perp line (line perpendicular to the mandibular plane Go-Me passing through Pog)
6-GoMe (mm)	Linear distance between the mesiovestibular cusp of the mandibular first molar perpendicular to GoMe

### Error study

Thirty lateral cephalograms were randomly selected, retraced, redigitized, and remeasured by the same examiner (L.S.N.) after a 30-day interval. Random and systematic errors were calculated by comparing the first and second measurements with Dahlberg's formula [15] and dependent t tests, respectively, at a significance level of 5%.

### Statistical analyses

Chi-square tests were used to check comparability among the three groups regarding sex distribution and severity of the initial class II molar relationship.

ANOVA followed by Tukey tests were used for intergroup comparison of the initial and final ages and cephalometric statuses, and treatment/observation changes.

Because the Bionator group (G2) had a significantly younger initial age and greater treatment time, the treatment changes were annualized according to the Jasper Jumper treatment time [14,16]. Therefore, all patients in G2 had their individual treatment changes, for each variable, divided by their treatment time, and then multiplied by the mean treatment time of G1.

All statistical analyses were performed with Statistica software (Statistica software for Windows, version 6.0, Statsoft, Tulsa, OK, USA.). Results were considered statistically significant at *P* < 0.05.

## Results

The random errors varied from 0.38 mm (1-GoMe) to 1.01 mm (6-ANSperp) and four variables (NAP, 6-ANSperp, 1-GoMe, and 6-GoMe) presented significant systematic errors.

The groups were comparable regarding sex distribution (Table 2).

**Table 2:** **Comparability among the groups regarding sex distribution (Chi-square test)**

**Gender**	**G1 Jasper (** ***n*** **= 25)**	**G2 Bionator (** ***n*** **= 30)**	**G3 Control (** ***n*** **= 22)**	***P***
Female	12 (48%)	14 (47%)	10 (45%)	0.9848 *χ* ^2^ = 0.0306
Male	13 (52%)	16 (53%)	12 (55%)	

Initial class II anteroposterior severity was significantly smaller in the control group, but similar between the experimental groups (Tables 3 and 4).

**Table 3:** **Comparability among groups regarding severity of the initial anteroposterior relationship of dental arches (Chi-square test)**

**Severity of class II**	**G1 Jasper (** ***n*** **= 25)**	**G2 Bionator (** ***n*** **= 30)**	**G3 Control (** ***n*** **= 22)**	***P***
¼ Class II	0	0	4 (18%)	0.0028 *χ* ^2^ = 19.9793
½ Class II	4 (16%)	9 (30%)	11 (50%)	
¾ Class II	9 (36%)	9 (30%)	4 (18%)	
Complete class II	12 (48%)	12 (40%)	3 (14%)

**Table 4:** **Compatibility between experimental groups in relation to initial severity of anteroposterior relationship of dental arches (Chi-square test)**

**Severity of class II**	**G1 Jasper (** ***n*** **= 25)**	**G2 Bionator (** ***n*** **= 30)**	***P***
¼ Class II	0	0	0.4769 *χ* ^2^ = 1.4808
½ Class II	4 (16%)	9 (30%)	
¾ Class II	9 (36%)	9 (30%)	
Complete class II	12 (48%)	12 (40%)	

The Bionator group had significantly younger initial age and greater treatment time than the other groups. The overjet at T1 was significantly greater in the Bionator than in the control group (Table 5).

**Table 5:** **Inter-group comparison of initial and final ages, observation interval, and overjet at T1 (ANOVA followed by Tukey tests)**

**Variables (in years)**	**G1 Jasper (** ***n*** **= 25)**		**G2 Bionator (** ***n*** **= 30)**		**G3 Control (** ***n*** **= 22)**		***P***
	**Mean**	**SD**	**Mean**	**SD**	**Mean**	**SD**	
Initial age	12.72A	1.21	11.31B	1.19	12.67A	0.75	0.0000*
Final age	14.88	1.20	15.23	1.17	14.80	1.71	0.4534
Observation interval	2.15A	0.30	3.92B	1.62	2.13A	1.64	0.0000*
Overjet at T1	7.38A,B	2.22	8.70A	2.58	5.86B	1.88	0.0002*

Different letters indicate statistically significant differences. *Statistically significant at *P* < 0.05.

The Bionator group had significantly smaller maxillary and mandibular length and posterior facial height than the other two groups, and had greater mandibular retrusion than the control group (Table 6). The maxillary incisors had significantly greater labial inclination in the Bionator than in the other groups whereas the mandibular incisors had significantly greater labial inclination in the Jasper Jumper than in the Bionator. The maxillary molars had a significantly greater mesial positioning in the Jasper Jumper group than in the other two groups. The Bionator group presented significantly smaller vertical development of the maxillary molars, mandibular incisors and mandibular molars than the Jasper Jumper group. Overjet was significantly greater in the Bionator than in the Jasper Jumper group.

**Table 6:** **Comparability at the pretreatment stage (T1) among the three groups (ANOVA followed by Tukey tests)**

**Variables**	**G1 Jasper (** ***n*** **= 25)**		**G2 Bionator (** ***n*** **= 30)**		**G3 Control (** ***n*** **= 22)**		***P***
	**Mean**	**SD**	**Mean**	**SD**	**Mean**	**SD**	
**Maxillary components**							
SNA (°)	82.58	3.38	82.15	2.92	81.65	3.29	0.6090
A-Nperp (mm)	1.34	3.63	0.09	3.05	0.69	2.55	0.3375
Co-A (mm)	85.87A	4.47	82.53B	3.54	87.01A	4.42	0.0005*
**Mandibular components**							
SNB (°)	77.21	2.56	76.11	2.80	77.54	3.67	0.1940
P-Nperp (mm)	−4.74A.B	5.01	−7.76A	5.54	−4.12B	4.15	0.0206*
Co-Gn (mm)	106.30A	5.13	100.22B	3.65	106.80A	5.81	0.0000*
**Maxillomandibular relationship**							
ANB (°)	5.38A.B	2.87	6.04A	2.09	4.11B	1.83	0.0147*
Wits (mm)	1.75A	2.52	1.43A,B	2.27	−0.16B	2.58	0.0206*
NAP (°)	8.97	7.30	10.18	5.31	6.91	4.70	0.1469
Growth pattern							
SN.GoGn (°)	31.12	4.01	32.95	5.75	30.83	4.58	0.2321
FMA (°)	24.72A,B	3.85	27.13A	4.77	24.17B	2.83	0.0186*
LAFH (mm)	61.81	4.22	59.49	4.64	60.70	3.95	0.1459
S-Go (mm)	69.34A	4.93	65.37B	4.55	69.65A	4.88	0.0019*
**Maxillary teeth**							
1.NA (°)	24.49A	7.30	29.69B	7.03	23.30A	6.02	0.0022*
1-NA (mm)	4.64	2.57	4.95	2.40	3.46	1.76	0.0670
1.PP (°)	114.48A	6.91	119.28B	6.96	113.11A	6.06	0.0030*
1-ANSperp (mm)	−1.85	2.64	−2.73	2.99	−3.56	2.56	0.1118
1-PP (mm)	26.51A,B	2.61	25.06A	2.16	26.58B	2.53	0.0367*
6-ANSperp (mm)	−30.65A	2.82	−32.98^B^	2.89	−32.57B	2.31	0.0060*
6-PP (mm)	20.95A	2.12	19.50B	1.90	20.57A,B	2.06	0.0256*
**Mandibular teeth**							
IMPA (°)	97.88A	7.52	92.62B	7.39	94.95A,B	4.71	0.0206*
1.NB (°)	28.65A	5.83	23.76B	7.33	25.66A,B	5.08	0.0196*
1-NB (mm)	5.10	2.06	3.67	2.53	3.98	1.80	0.0511
1-GoMe (mm)	38.63A	2.84	36.12B	2.60	37.20A,B	2.40	0.0031*
6-Pperp (mm)	−29.21	2.19	−29.88	1.98	−30.15	2.09	0.2768
6-GoMe (mm)	27.91A	2.31	25.76B	1.93	27.45A	2.10	0.0007*
**Dental relationships**							
Overjet (mm)	6.24A	2.21	8.42B	2.93	4.70A	1.60	0.0000*
Overbite (mm)	4.94	1.68	4.64	2.25	4.62	1.71	0.8011
Molar relationship (mm)	−1.38A	1.15	−0.98A	1.22	0.69B	1.23	0.0000*

Different letters indicate statistically significant differences. *Statistically significant at *P* < 0.05.

Both appliances presented similar changes regarding maxillary and mandibular components, maxillomandibular relationship, maxillary and mandibular teeth, and in overjet and overbite, except for the maxillary molars that had significantly greater distalization in the Jasper Jumper group than in the Bionator and control groups (Table 7). Molar relationship had significantly greater improvement in the Jasper Jumper than in the Bionator group, and both had greater improvements than the control group (Table 7).

**Table 7:** **Intergroup comparison of treatment and growth changes (T2-T1) (ANOVA followed by Tukey tests)**

**Variables**	**G1 Jasper (n = 25)**		**G2 Bionator (n = 30)**		**G3 Control (n = 22)**		**P**
	**Mean**	**SD**	**Mean**	**SD**	**Mean**	**SD**	
**Maxillary Components**							
SNA (°)	−1.23A	2.09	−0.64A	1.26	0.90B	2.56	0.0014*
A-Nperp (mm)	−1.26A	2.96	−0.27A	1.21	1.53B	3.01	0.0009*
Co-A (mm)	0.61A	2.39	1.91A,B	1.59	2.63B	3.07	0.0141*
**Mandibular Components**							
SNB (°)	0.09	0.96	0.85	0.95	0.62	2.07	0.1261
P-Nperp (mm)	−0.10	4.22	1.89	2.50	2.39	4.65	0.0579
Co-Gn (mm)	4.05	2.81	5.01	2.66	4.45	4.39	0.5536
**Maxillomandibular Relationship**							
ANB (°)	−1.32A	1.58	−1.49A	1.34	0.29B	1.21	0.0000*
Wits (mm)	−1.16A	2.29	−0.12A	1.58	1.18B	1.97	0.0005*
NAP (°)	−3.06A	3.69	−3.38A	3.02	0.21B	2.66	0.0002*
Growth Pattern							
SN.GoGn (°)	0.57A	1.50	−0.28A	1.36	−0.43A	1.72	0.0483*
FMA (°)	0.72A	2.54	−0.58B	1.35	−1.07B	2.01	0.0076*
LAFH (mm)	3.63A	2.03	2.49A,B	1.24	2.06B	2.88	0.0302*
S-Go (mm)	3.70	2.36	4.32	2.00	2.75	3.58	0.1154
**Maxillary Teeth**							
1.NA (°)	−2.11	8.48	−3.64	6.04	−1.08	2.28	0.3352
1-NA (mm)	−0.88	2.83	−0.83	1.61	−0.01	1.36	0.2668
1.PP (°)	−2.95	7.79	−3.67	6.02	0.15	2.26	0.0657
1-ANSperp (mm)	−2.02A	2.27	−0.90A	1.57	0.94B	1.97	0.0000*
1-PP (mm)	1.48A	1.21	1.05A,B	1.06	0.67B	0.98	0.0455*
6-ANSperp (mm)	−0.73A	2.12	0.61B	1.48	0.67B	1.75	0.0093*
6-PP (mm)	0.97	1.24	1.28	0.96	1.69	1.30	0.1121
**Mandibular Teeth**							
IMPA (°)	2.26	5.78	4.90	9.53	0.08	3.56	0.0551
1.NB (°)	2.92A.B	5.44	5.55A	10.11	0.28B	4.27	0.0450*
1-NB (mm)	1.56A,B	1.39	1.63A	2.29	0.41B	1.57	0.0420*
1-GoMe (mm)	0.16A	1.45	1.06A,B	1.03	1.38B	2.14	0.0216*
6-Pperp (mm)	0.82A	1.13	0.37A,B	1.00	−0.30B	1.32	0.0048*
6-GoMe (mm)	3.00A	1.14	2.29A	1.06	1.09B	1.93	0.0001*
**Dental relationships**							
Overjet (mm)	−3.73A	2.29	−3.89A	4.16	0.16B	1.24	0.0000*
Overbite (mm)	−2.84A	1.36	−1.77A	2.05	−0.25B	2.11	0.0001*
Molar relationship (mm)	3.42A	1.16	2.19B	1.83	−0.18C	1.30	0.0000*

Different letters indicate statistically significant differences. *Statistically significant at *P* < 0.05.

## Discussion

When comparing treatment and growth changes of the three groups, it was observed that there is a restriction of maxillary forward displacement in both experimental groups compared to the control group (Table 7). Regarding the effective length of the maxilla (Co-A), there was a statistically significant restriction of maxillary growth between G1 and the control group; however, restriction of maxillary growth of G2 was similar to G1 and the control group. These results agree with previous studies that also found significant restrictions of maxillary growth during Jasper Jumper [10,11,14,17] and Bionator therapies [18,19].

Regarding the mandibular components, none of the evaluated variables presented statistically significant differences when comparing the three groups (Table 7). Results in the variables related to mandibular position (SNB and P-Nperp) are probably due to clockwise rotation of the mandible that occurred during treatment causing an increase in LAFH in both experimental groups. Changes in mandibular effective length (Co-Gn) in both experimental groups are probably inherent to normal growth. Previous studies reported significant protrusion of the mandible after treatment with the Jasper Jumper [11,17], whereas other studies did not show any significant changes in the growth or position of the mandible [2,12]. Regarding treatment with the Bionator, some authors observed an increase in the protrusion or effective length of the mandible [16,20–22]. However, another study revealed no changes in mandibular protrusion or increments during treatment with the Bionator [23]. In addition, authors who have studied the skeletal effects of other functional appliances also showed no significant effects on the mandible [24,25].

There was significant improvement in the maxillomandibular relationship of the experimental groups compared to the control group (Table 7). In fact, the improvement in the relationship between maxilla and mandible seems to have occurred as a result of the restriction of anterior displacement of the maxilla in the experimental groups, associated with normal growth of the mandible. Supporting these results, several authors have reported improvements in the maxillomandibular relationship after treatment with the Jasper Jumper [2,17] and the Bionator [21].

Treatment with the Jasper Jumper associated with fixed appliances showed a mild, but significant clockwise rotation of the mandible and an increase in LAFH, denoting a vertical influence of this protocol on facial structures (Table 7). Treatment with the Bionator followed by fixed orthodontic appliance did not cause significant vertical changes when compared to the control group. The experimental groups showed different behaviors in variables related to growth pattern. There was clockwise rotation of the mandible in patients treated with the Jasper jumper, whereas patients treated with the Bionator showed the same trend of the control group where the mandible presented a counterclockwise rotation. Previous studies [10,17] also observed the same rotation of the mandible as a result of treatment with the Jasper Jumper, while others reported that treatment with the Bionator does not significantly change the growth pattern variables, as reported here [20,21]. In contrast, some studies [2,11] revealed no significant vertical changes due to treatment with the Jasper Jumper and others [22,26] observed an increase of the vertical dimensions after treatment with the Bionator. Therefore, it seems that vertical changes with these appliances are similar.

The maxillary incisors had significantly greater retrusion and extrusion in the experimental groups (Table 7). Previous studies [10-12,17] also observed retrusion of the maxillary incisors in patients treated with the Jasper Jumper, while other researches [10,12,17] indicated extrusion of the maxillary incisors using Jasper Jumper. Probably, palatal tipping was minimized in the Jasper Jumper group by the incorporation of labial crown torque in the maxillary incisors. Other studies also observed all these dentoalveolar effects on maxillary incisors during Bionator treatment [20,21,23].

The maxillary molars had significantly greater distalization with the Jasper Jumper when compared to the other groups (Table 7). These distalizing effects of the maxillary molars were already reported by several authors when using the Jasper Jumper [10-12,17]. No significant difference between the groups was observed in relation to the vertical changes of the maxillary molars (6-PP) (Table 7). Probably, there was restriction of the vertical development of the maxillary molars during the orthopedic phase in the Jasper Jumper and Bionator groups; however, these teeth may have been extruded during the fixed mechanical correction. Therefore, it becomes difficult to differentiate the dental effects caused by orthopedic and orthodontic appliances.

The mandibular incisors had significantly greater labial tipping in the Bionator than in the control group and significantly greater protrusion and intrusion in the experimental compared to the control group (Table 7). These effects are in accordance with previous studies which pointed protrusion of these teeth using the Jasper Jumper [10–12,17] as well as its intrusion [10,12,17]. Labial tipping in the Jasper Jumper was not statistically significant compared to the control group. This effect was probably minimized due to the lingual torque which was incorporated in the rectangular archwire in the mandibular anterior teeth [14]. It is well known that the forces applied by fixed functional appliances in class II treatment have an intrusive vector in the mandibular incisors. Thus, the significant limitation of the vertical development of the mandibular incisors observed in the Jasper Jamper group compared to the control group is due to the intrusive effect of this appliance in the anterior region. Labial tipping of mandibular incisors in patients treated with the Bionator was previously described in the literature [2,18,20–23,26], whereas one study [22] reported that these teeth had no significant changes during treatment with this appliance.

The mandibular molars had significantly greater mesial movement in the Jasper Jumper when compared to the control group. Additionally, significantly greater extrusion was observed in the experimental groups (Table 7). These results agree with most of the studies which also reported mesialization and extrusion of the mandibular molars [10-12,14,17] with the use of Jasper Jumper appliance. This greater extrusion of mandibular molars in the Bionator group compared to the control group is also in agreement with previous studies in the literature [18,20-22]. This increased vertical development of the mandibular molars was expected since acrylic trimming is performed with this objective, based on Harvold's principle of differential eruption, depending on the need to correct the overbite that normally accompanies class II, division 1 malocclusion [13,14].

The significantly greater improvements in the experimental groups regarding dental relationships are due to dental and skeletal changes described above (Table 7). Several authors have already demonstrated these corrections using the Jasper Jumper [10–12,14,17], as well as with the Bionator [19–22].

Although the results of this study are supported by several studies in the literature, a randomized clinical trial could provide stronger scientific evidence. However, obtaining a control group would be very difficult due to ethical reasons.

## Conclusions

The effects of the Jasper Jumper and the Bionator appliances followed by fixed orthodontic appliances were basically similar in class II malocclusion treatment. Their main effects are as follows:

1. A restrictive effect on the maxilla;
2. A slight increase in anterior face height;
3. Retrusion and extrusion of the maxillary incisors;
4. Labial tipping and protrusion of the mandibular incisors in both groups and intrusion with the Jasper Jumper appliance;
5. Maxillary molar distalization with the Jasper Jumper;
6. Extrusion and mesialization of the mandibular molars;
7. Both appliances provided significant improvement of maxillomandibular relationship, overjet, overbite and molar relationship.
